# Early Growth Response Genes 2 and 3 Regulate the Expression of *Bcl6* and Differentiation of T Follicular Helper Cells*

**DOI:** 10.1074/jbc.M114.634816

**Published:** 2015-05-15

**Authors:** Ane Ogbe, Tizong Miao, Alistair L. J. Symonds, Becky Omodho, Randeep Singh, Punamdip Bhullar, Suling Li, Ping Wang

**Affiliations:** From the ‡ Division of Biosciences, Department of Life Sciences, Brunel University, Kingston Lane, UB8 3PH, United Kingdom and; the § Blizard Institute of Cell and Molecular Science, Barts and London School of Medicine and Dentistry, Queen Mary University of London, 4 Newark Street, London E1 2AD, United Kingdom

**Keywords:** antibody, cellular immune response, T helper cells, transcription factor, viral immunology

## Abstract

T follicular helper (Tfh) cells support differentiation of B cells to plasma cells and high affinity antibody production in germinal centers (GCs), and Tfh differentiation requires the function of B cell lymphoma 6 (BCL6). We have now discovered that early growth response gene 2 (EGR2) and EGR3 directly regulate the expression of *Bcl6* in Tfh cells, which is required for their function in regulation of GC formation. In the absence of EGR2 and -3, the expression of BCL6 in Tfh cells is defective, leading to impaired differentiation of Tfh cells, resulting in a failure to form GCs following virus infection and defects in production of antiviral antibodies. Enforced expression of BCL6 in EGR2/3-deficient CD4 T cells partially restored Tfh differentiation and GC formation in response to virus infection. Our findings demonstrate a novel function of EGR2/3 that is important for Tfh cell development and Tfh cell-mediated B cell immune responses.

## Teaser

**Background:** T follicular helper (Tfh) cells are essential for B cell responses against viruses and require B cell lymphoma 6 (BCL6).

**Results:** Early growth response gene 2 (EGR2) and EGR3 intrinsically regulate *Bcl6* and Tfh cells.

**Conclusion:** EGR2 and -3 are novel regulators of BCL6-mediated Tfh development.

**Significance:** In addition to controlling inflammation, EGR2 and -3 regulate Tfh function during antiviral immunoresponses.

## Introduction

Humoral immunity depends on help provided by T follicular helper (Tfh)^4^ cells, which support the differentiation of antigen-specific B cells into memory and plasma cells in germinal centers (GCs) (1, 2). Recently, it has been proposed that Tfh differentiation consists of two stages, the induction of CXCR5 expression in the T cell zone and the expression of BCL6, a transcription factor that is essential for Tfh differentiation (2), after migration into the interfollicular and follicular regions (3). BCL6 has been discovered to be a master regulator of Tfh differentiation (4–6). BCL6 is expressed in both Tfh and GC B cells and is autonomously required for the function of both cells (7–11). Although it is necessary for Tfh cell development, the mechanism of BCL6 function in Tfh differentiation is still poorly understood. BLIMP1 is a negative regulator of Tfh differentiation through transcriptional repression of the *Bcl6* gene (4). In addition to BCL6, the transcription factors c-MAF, BATF, and IRF4 have been found to regulate Tfh cell differentiation (12–15) by regulating either *Bcl6* expression (13) or STAT3-mediated IL-21 expression (15, 16). Moreover, SH2D1A and ICOS are required for Tfh cell migration into the GC area and are essential for GC formation (12, 17).

Early growth response gene 2 (EGR2) and EGR3, members of the EGR transcription factor family, are important for the control of inflammatory autoimmunity and antigen receptor-mediated lymphocyte proliferation (18, 19). Although EGR2 and -3 are expressed in effector phenotype T cells, their roles in regulation of T cell effector function are still not fully understood. Analysis of the global gene expression patterns of EGR2/3-deficient T cells revealed that in addition to inflammatory cytokines (19), the expression of the Tfh regulator *Bcl6* was significantly reduced, whereas *Prdm1* (BLIMP1), a functional antagonist of BCL6 and Tfh differentiation, was increased. We found that the differentiation of Tfh cells in *CD2-Egr2*^−/−^*Egr3*^−/−^mice, which lack EGR2 and -3 in B and T cells (19), was severely defective, resulting in failure to form GCs after virus infection. EGR2 directly controlled the expression of *Bcl6* in Tfh cells, whereas enforced expression of BCL6 in EGR2/3-deficient CD4 T cells effectively restored GC formation. Thus, we have discovered a novel function of EGR2/3 in regulation of *Bcl6* expression, Tfh differentiation, and GC development.

## Experimental Procedures

### 

#### 

##### Mice

*Egr3*^−/−^ and CD2-specific *Egr2*^−/−^ (K2/3) mice on the C57BL/6 background were described in our previous report (19). C57BL/6 mice were used as controls in all experiments. *Rag2*^−/−^ mice on a C57BL/6J background were bred in-house and described in our previous report (19). All mice were maintained in the Biological Services Unit, Brunel University, and used according to established institutional guidelines under the authority of a UK Home Office project license.

##### Antibodies and Flow Cytometry

Fluorescein isothiocyanate (FITC)-conjugated antibodies to B220, PD1, GL7, and CD4; eFluor 450-labeled antibody to CD4; phycoerythrin-conjugated antibodies to CD4, CD19, CD25, PD1, CD62L, CD69, IgM, EGR2, and BCL6; PerCP-labeled antibody to CXCR5 and B220; phycoerythrin-Cy7-labeled antibody to B220; biotin-conjugated antibody to CXCR5; allophycocyanin-conjugated antibodies to CD44 and EGR2, and allophycocyanin-labeled streptavidin were obtained from eBioscience. For flow cytometry analysis, single cell suspensions were analyzed on a Canto II system (BD Immunocytometry Systems), and the data were analyzed using FlowJo (Tree Star). Cell sorting was performed on a FACSAria sorter with DIVA option (BD Immunocytometry Systems).

##### Cell Isolation and Stimulation

Naive CD4^+^ T and B cells were purified by negative selection using MACS systems (Miltenyi Biotec). CD4^+^PD1^+^CXCR5^+^ cells were sorted by FACS after staining with FITC-conjugated PD1, phycoerythrin-conjugated CD4, and PerCP-conjugated CXCR5 antibodies. Purified CD4^+^ T cells were stimulated with plate-bound anti-CD3 (clone 145-2C11, BD Biosciences) at 5 μg/ml or at the indicated concentrations and anti-CD28 (clone 37.51, BD Bioscience) (2 μg/ml) antibodies for the indicated times. B cells were activated with soluble anti-F(ab′)_2_ fragments of goat anti-mouse IgM (anti-IgM F(ab′)_2_, Jackson ImmunoResearch Laboratories, Inc.) at 5 μg/ml for the indicated times.

##### Microarray Analysis

Naive CD4 T cells were stimulated with 5 μg/ml anti-CD3 and 2 μg/ml anti-CD28 for 16 h or left unstimulated before extraction of total RNA. 250 ng of total RNA was processed using the Ambion WT expression and Affymetrix GeneChip WT terminal labeling kits according to the manufacturers' instructions. The labeled cDNA was then hybridized to Mouse Gene 1.0 ST arrays (Affymetrix) using the GeneChip hybridization, wash, and stain kit (Affymetrix) and scanned to generate .CEL files.

Array data were analyzed using R (20). Data were normalized and summarized at the “core genes” level using the robust multiarray average method as implemented in the Bioconductor oligo package (21). The data were filtered to remove transcripts that had more than 50% of probe sets with a detection above background *p* value greater than 0.05 and transcripts with cross-hybridizing probe sets. Scatter plots were created using the ggplot2 package (22).

For the clustered heat map, the data were filtered for transcripts with expression values greater than 120 in any sample, and probe sets associated with the same gene symbol were consolidated by selection of the probe set with the highest mean expression. Data were “row-centered” by subtraction of the mean expression level for each transcript, and transcripts annotated with the GO term GO:0005125 (cytokine activity) were selected. The final heat map was generated using a hierarchical clustering algorithm, with relative expression levels represented by a relative color scale, using the gplots package (23). Microarray data are available from the ArrayExpress database under accession number E-MTAB-2432.

##### Quantitative Real-time PCR

Total RNA was extracted from stimulated or unstimulated CD4^+^ T cells or from CD4^+^PD1^+^CXCR5^+^ cells, using an RNeasy kit (Qiagen) or TRIzol (Invitrogen) and reverse transcribed using oligo(dT) primers (Amersham Biosciences). Quantitative real-time PCR was performed on a Rotor-Gene system (Corbett Robotics) using SYBR Green PCR master mix (Qiagen). Primers used in PCR were as follows: *IL21*, 5′-CTCAAGCCATCAAACCCTGG-3′ (sense) and 5′-CATACGAATCACAGGAAGGG-3′ (antisense); *Bcl6*, 5′-CATGCAGGAAGTTCATCAAGG-3′ (sense) and 5′-CTCAGTGGCATATTGTTCTCC-3′ (antisense); *Cxcr5*, 5′-TATGGATGACCTGTACAAGGA-3′ (sense) and 5′-AGGATGTTTCCCATCATACCC-3′ (antisense); *Icos*, 5′-CAAGAAAGGAACCTTAGTGGA-3′ (sense) and 5′-CACTATTAGGGTCATGCACAC-3′ (antisense); *Irf4*, 5′-CAGCTCATGTGGAACCTCTG-3′ (sense) and 5′-TTGTTGTCTTCAAGTGGAAACCC-3′ (antisense); *Prdm1*, 5′-AACCTGAAGGTCCACCTGAG-3′ (sense) and 5′-TGCTAAATCTCTTGTGGCAGAC-3′ (antisense); *Ascl2*, 5′-ACTGTCTAGAACTTTCCAACC-3′ (sense) and 5′-AAACATCAGCGTCAGTATAGG-3′ (antisense); *Egr2*, 5′-CTTCAGCCGAAGTGACCACC-3′ (sense) and 5′-GCTCTTCCGTTCCTTCTGCC-3′ (antisense); *Ifng*, 5′-CCATCAGCAACAACATAAGC-3′ (sense) and 5′-AGCTCATTGAATGCTTGGCG-3′ (antisense); *Sh2d1a*, 5′-CCTGTAATAGCATCTCGCCTGAT-3′ (sense) and 5′-AGTTTTCCAATCCGCACTTTAAAG-3′ (antisense).

The data were analyzed using the Rotor-Gene Software. All samples were run in duplicate, and relative mRNA expression levels were obtained by normalizing against the level of β-actin from the same sample under the same program using relative expression = 2̂(CTβ-actin-CT target) × 10,000.

##### ChIP Assays

ChIP assays were performed according to the protocol supplied with the kit (catalog no. 9003) from Cell Signaling Technology. Briefly, 5 × 10^7^ CD4 cells from wild type mice were stimulated with anti-CD3 and anti-CD28 for 16 h. The cells were then cross-linked with 1% formaldehyde for 10 min at room temperature. After quenching of formaldehyde with 125 mm glycine, chromatin was sheared by sonication. The fragmented chromatin was around 300–1000 bp as analyzed on agarose gels. After preclearing, chromatin (500 μg) was subjected to immunoprecipitation with specific anti-EGR2 antibody (eBioscience) or with anti-Ig as negative control at 4 °C overnight. Immunocomplexes were recovered by incubation with blocked protein G beads. DNA was purified according to the kit and used as template for PCR with specific primers: *Bcl6* 1 (intron 1), 5′-GAAGATGAACTGGATTCCTCCC-3′ (sense) and 5′-CCCTCAAAGCTCTTAACCGA-3′ (antisense); *Bcl6* 2 (promoter), 5′- AAAGGTGAATACAGGGCAGAC-3′ (sense) and 5′-GAAACAAGAGTCTCACTCATCC-3′ (antisense); *Bcl6* 3 (downstream), 5′-TGAATCACGGATGCATAAATGG-3′ (sense) and 5′-TGACCGACAGACATTCACAG-3′ (antisense).

##### EMSA

The consensus probe for EGR2 (5′-TGTAGGGGCGGGGGCGGGGTTA-3′) was labeled with Cyanine5.5 (Sigma-Aldrich) and used in binding reactions with nuclear extracts from CD4 T cells stimulated with anti-CD3 and anti-CD28 for 16 h and then restimulated for 30 min with phorbol 12-myristate 13-acetate and ionomycin (18, 19). For supershift reactions, anti-EGR2 (eBioscience) was added after 10 min of incubation. The samples were electrophoresed on 5% polyacrylamide gels in 0.5× TBE. The gels were scanned using an Odyssey Imager (LI-COR). For competition assays, oligonucleotides containing the three EGR2 binding sites from the Bcl6 locus, identified using Mulan (24) (*Bcl6*-1 (Chr16, 23983443–23983464) 5′-GGAGGGGGCGGGGGAGACAGCT-3′; *Bcl6*-2 (Chr16, 23990225–23990248) 5′-TTGCCCTCCTACTCATCCCTGGAT-3′; and *Bcl6*-3 (Chr16, 23964461–23964482) 5′-ACACAGGAGGAGGTGGCTGAGT-3′) were added into the reaction mixtures prior to incubation.

##### Lentiviral Transduction

The lentiviral constructs for *Egr2* or *Bcl6* were constructed by PCR cloning. *Egr2* was transferred from an expression construct (18). Primers for *Egr2* cloning were 5′-ACTCAGATCTCGAGGCCACCATGGACTACAAAGACGATGACGACAAGACCGCCAAGGCCGTAGAC-3′ (sense) and 5′-AGCTAGCTAGCGAGAATTCCTACAATTCCGG-3′ (antisense), and those for *Bcl6* were 5′-AAGCTGGCTAGCGCCGCCATGGCCTCCCCGGCTGAC-3′ (sense) and 5′-AGGGGCGGATCCTCAGCAGGCTTTGGGGAGC-3′ (antisense). The constructs were confirmed by sequencing. In addition to *Egr2* or *Bcl6*, the constructs carry an internal ribosome entry site-driven GFP, which allows us to isolate transduced cells by fluorescence-activated cell sorting. Naive CD4 cells from *CD2-Egr2*^−/−^*Egr3*^−/−^ mice at 1 × 10^6^ cells/well in a 24-well plate coated with anti-CD3 and anti-CD28 were infected with concentrated lentivirus at a multiplicity of infection of 50–100 (∼10^5^ to 10^6^ transducing units/ng of p24) as described previously (19). The infected cells were incubated at 37 °C for 7 h with gentle shaking before the addition of 1 ml of medium. The cells were harvested after 24 h, and the GFP-positive cells were isolated by cell sorting.

##### Viruses

VV_WR_ stocks were grown using TK143 cells in T175 flasks, infected at a multiplicity of infection of 0.5. Cells were harvested at 72 h, and virus was isolated by rapidly freeze-thawing the cell pellet three times in 5 ml of DMEM containing 10% fetal calf serum (FCS) as described previously (25). Cell debris was removed by centrifugation. Clarified supernatant was frozen at −80 °C as virus stock. VV_WR_ stocks were titrated using TK143 cells.

##### Viral Infection

Mice were infected intranasally with 2 × 10^5^ pfu of vaccinia virus in 10 μl of physiological saline. The mice were weighed and observed for illness daily, as described previously (25). *In vivo* replication of vaccinia virus was examined by plaque assay on lung tissue samples, which were removed, weighed, and ground with a mortar and pestle. Serial 10-fold dilutions of clarified supernatants were used to infect subconfluent monolayers of TK143 cells in triplicate in 24-well plates. The cells were fixed with formalin 2 days after infection and stained with 2% crystal violet in 40% methanol, and plaques were counted under a dissecting microscope.

##### Plaque Reduction Neutralization Tests

TK143 cells were seeded into 24-well Costar plates (Corning Inc.) and used within 2 days of reaching confluence. Sera were diluted in DMEM. The serially diluted sera were then incubated with an equal volume of VV_WR_ (2 × 10^4^ pfu/ml) overnight at 37 °C. The cells were rinsed in serum-free medium, the medium was aspirated, and 100 μl of virus/serum mixture was added to each well in duplicate and left to adsorb for 60 min at 37 °C with periodic swirling. The wells were then washed with serum-free medium, and normal growth medium was added. After allowing 2 days for the plaques to develop, the cells were fixed and stained in one step with 0.1% crystal violet in 20% ethanol, and the plaques were quantified over white light transillumination.

##### Adoptive Transfer

2 × 10^6^ naive CD4^+^ T or resting B cells from 4-week-old wild type mice were suspended in 100 μl of physiological saline and injected intravenously into the dorsal tail vein of 6-week-old *CD2-Egr2*^−/−^*Egr3*^−/−^ mice. 24 h after transfer, the recipient mice were infected intranasally with 2 × 10^5^ pfu of vaccinia virus. 14 days after infection, Tfh and GC B cells and GC formation were analyzed. For *Rag2*^−/−^ experiments, either (*a*) 2.5 × 10^6^ naive wild type CD4^+^ T and 2.5 × 10^6^ resting wild type B cells or (*b*) 2.5 × 10^6^ naive wild type CD4^+^ T and 2.5 × 10^6^ resting B cells from *CD2-Egr2*^−/−^*Egr3*^−/−^ mice or (*c*) 2.5 × 10^6^ resting wild type B cells and 2.5 × 10^6^ naive CD4 cells from *CD2-Egr2*^−/−^*Egr3*^−/−^ mice were suspended in 100 μl of physiological saline and injected intravenously into the dorsal tail vein of *Rag2*^−/−^ mice. Forty days after transfer, recipient mice were infected intranasally with 2 × 10^5^ pfu of vaccinia virus. For adoptive transfer of transduced cells, 1 × 10^6^ EGR2- or BCL6-transduced EGR2/3-deficient CD4 cells were suspended in 100 μl of physiological saline and injected intravenously into the dorsal tail vein of *CD2-Egr2*^−/−^*Egr3*^−/−^ mice. Tfh and GC B cells and GC formation were analyzed 14 days after infection.

##### Generation of Bone Marrow Chimeras

Bone marrow was collected from *CD2-Egr2*^−/−^*Egr3*^−/−^ (CD45.2^+^) and wild type C57BL/6 (CD45.1^+^) mice. For each chimera, 20 × 10^6^ cells of a 1:1 mixture of *CD2-Egr2*^−/−^*Egr3*^−/−^ and C57BL/6 bone marrow cells were transferred intravenously into lethally irradiated (two doses of 550 rads) wild type C57BL/6 (CD45.1^+^) recipients. Recipient mice were allowed 8 weeks to recover following reconstitution before intranasal infection with 2 × 10^5^ pfu of vaccinia virus. 14 days after infection, Tfh and GC B cells among gated CD45.1^+^ and CD45.2^+^ cells were analyzed.

##### Immunohistochemistry Analysis

Spleen tissue sections were fixed with 4% paraformaldehyde in PBS and embedded in paraffin or by 1:1 acetone/methanol fixation for frozen sections. Paraffin sections were stained with hematoxylin and eosin. Histological examination of tissue sections was done in a blind manner. Frozen sections were stained with TRITC-conjugated peanut agglutinin (PNA; Sigma), Alexa Fluor 647-labeled anti-IgD (Biolegend), and FITC-labeled anti-CD4 mAb (BD Biosciences). Paraffin sections were stained using rat anti-mouse B220 (BD Biosciences), which was detected using anti-rat FITC-labeled IgG (Sigma), and rabbit anti-CD3 (DAKO), which was detected using anti-rabbit Alexa Fluor 647-labeled IgG (Invitrogen), together with TRITC-conjugated PNA (Sigma). Stained sections were washed in phosphate-buffered saline (PBS) and mounted using Vectashield (Vector Laboratories, Burlingame, CA).

##### Statistics

Graphs were generated using the using the R package ggplot2 (22). A two-tailed non-parametric Mann-Whitney test was used to analyze the statistical significance of differences between groups using the R package coin (26). Differences with a *p* value of <0.05 were considered significant.

## Results

### 

#### 

##### Altered Expression of BCL6 and BLIMP1 in EGR2/3-deficient CD4 T Cells

**FIGURE 1.**
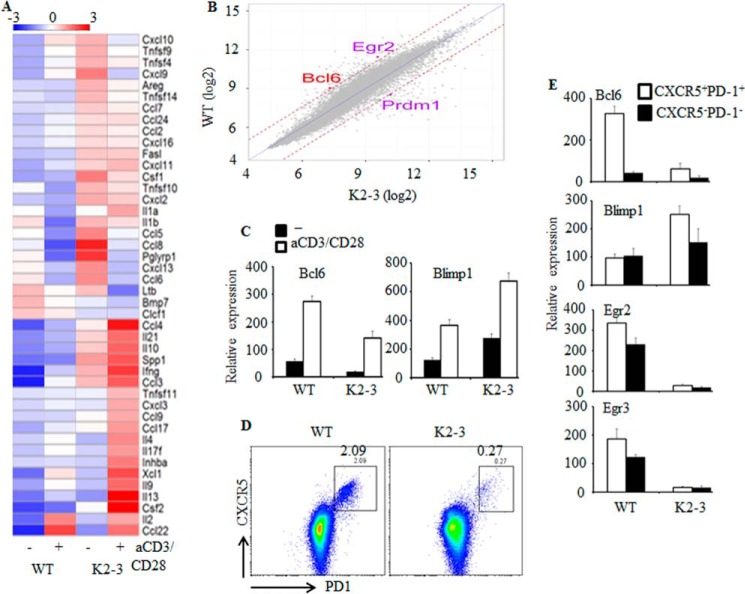
**Altered expression of Tfh cell genes in EGR2/3-deficient CD4 T cells.** *A*, heat map presentation of differentially expressed cytokine genes (GO term GO:0005125) from unstimulated (−) and anti-CD3- and anti-CD28-stimulated (+) CD4 T cells from WT and *CD2-Egr2*^−/−^*Egr3*^−/−^ (K2-3) mice. *B*, scatter plot of gene expression levels for anti-CD3- and anti-CD28-stimulated CD4 T cells from WT and K2-3 mice. *Dotted blue line*, equivalent expression between samples; *dashed brown lines*, 3-fold differential expression between WT and K2-3. *Bcl6*, *Egr2*, and *Prdm1* (BLIMP1) are highlighted. *C*, differential expression of *Bcl6* and BLIMP1 was confirmed by RT-PCR using unstimulated and anti-CD3- and anti-CD28-stimulated CD4 T cells from WT and K2-3 mice. *D*, CXCR5^+^PD1^+^ cells from WT and K2-3 mice were analyzed after gating on the CD4 population. *E*, relative expression of the indicated genes in CD4^+^CXCR5^+^PD1^+^ and CD4^+^CXCR5^−^PD1^−^ cells. RT-PCR results in *C* and *E* are presented relative to the expression of β-actin mRNA. Data in *C* and *E* represent three independent experiments.

EGR2 and -3 have overlapping functions in T cell receptor-mediated responses (19). To assess the mechanisms, we analyzed the global gene expression patterns of EGR2- and EGR3-deficient CD4 T cells. CD4 T cells were isolated from wild type and *CD2-Egr2*^−/−^*Egr3*^−/−^ mice at 2 months of age and stimulated with anti-CD3 and anti-CD28 for 16 h, and the global gene expression patterns of these cells were analyzed. A total of 482 genes were found to be differentially expressed, using a threshold of 3-fold, in EGR2- and EGR3-deficient CD4 T cells compared with wild type counterparts (ArrayExpress accession number E-MTAB-2432). Among the genes that were increased in EGR2- and EGR3-deficient T cells, there was a large number of inflammatory cytokines and chemokines (Fig. 1*A*), consistent with our previous findings of increased inflammatory responses of EGR2- and EGR3-deficient T cells (19). Interestingly, among the genes that were differentially expressed in EGR2- and EGR3-deficient CD4 cells were some of the key regulators of Tfh cells (Fig. 1*B*). The expression of *Bcl6* was reduced, whereas, in contrast, BLIMP1 (encoded by *Prdm1*), a repressor of *Bcl6*, was increased in EGR2/3-deficient CD4 T cells (Fig. 1*B*). The altered expression of *Bcl6* and BLIMP1 was confirmed by real-time PCR (Fig. 1*C*). Notably, despite the defects in *Bcl6* expression in EGR2- and EGR3-deficient CD4 T cells, a low level of *Bcl6* could still be induced in response to T cell receptor stimulation (Fig. 1*C*), indicating that EGR2 and -3 are not the only transcription factors that regulate *Bcl6* expression. To analyze the expression of these genes in Tfh cells, CD4^+^CXCR5^+^PD1^+^ cells (Fig. 1*D*) were isolated from *CD2-Egr2*^−/−^*Egr3*^−/−^ and wild type mice and analyzed for expression of *Bcl6* and BLIMP1 by real-time PCR. Notably, CD4^+^CXCR5^+^PD1^+^ cells were significantly reduced in *CD2-Egr2*^−/−^*Egr3*^−/−^ mice (Fig. 1*D*). The expression of *Bcl6* was severely defective in EGR2- and EGR3-deficient Tfh cells, whereas BLIMP1 expression was increased (Fig. 1*E*).

**FIGURE 2.**
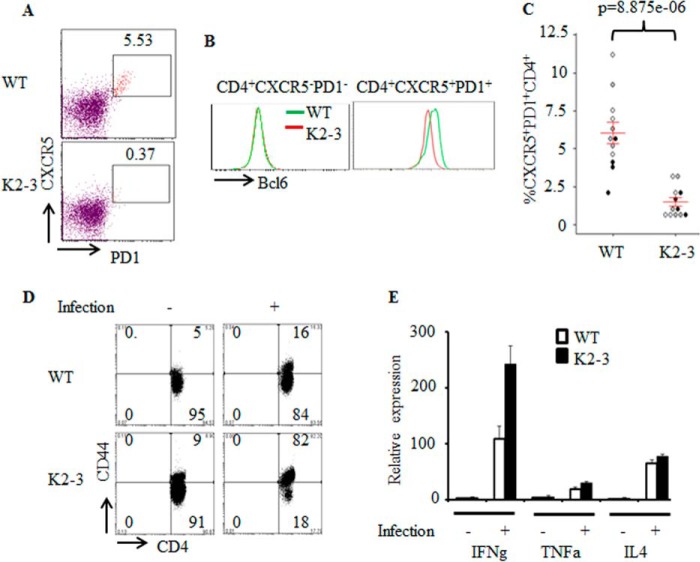
**Impaired Tfh differentiation in *CD2-Egr2*^−/−^*Egr3*^−/−^ mice.** WT and *CD2-Egr2*^−/−^*Egr3*^−/−^ (K2-3) mice were infected intranasally with 2 × 10^5^ pfu of vaccinia virus. *A* and *C*, Tfh cells in WT and K2-3 mice 14 days postinfection were analyzed by flow cytometry using the indicated markers after gating on the CD4^+^ population (*A*), and CXCR5^+^PD1^+^ cells were quantified as a percentage of total CD4^+^ cells (*C*). *B*, BCL6 expression by CD4^+^CXCR5^+^PD1^+^ and CD4^+^CXCR5^−^PD1^−^ cells in WT and K2-3 mice 14 days postinfection was analyzed by flow cytometry. *D* and *E*, CD4 T cells from WT and K2-3 mice 7 days postinfection were analyzed for expression of CD44 by flow cytometry (*D*) and production of effector cytokines by RT-PCR (*E*). In *C*, each *symbol* represents an individual mouse from three independent experiments (indicated by *different colored symbols*); *error bars*, S.E. on either side of the mean. *, *p* < 0.0005 (Mann-Whitney two-tailed test). *E*, mRNA samples were pooled from four mice, and RT-PCR results are presented relative to the expression of β-actin mRNA. Data are representative of at least two independent experiments.

To assess the consequences of defective *Bcl6* expression in Tfh cells in *CD2-Egr2*^−/−^*Egr3*^−/−^ mice, we analyzed Tfh cells after vaccinia virus infection. We discovered that the differentiation of Tfh cells in response to virus infection was defective in *CD2-Egr2*^−/−^*Egr3*^−/−^ mice compared with wild type counterparts (Fig. 2, *A* and *C*). Consistently, the expression of BCL6 in CD4^+^CXCR5^+^PD1^+^ cells from *CD2-Egr2*^−/−^*Egr3*^−/−^ mice was reduced (Fig. 2*B*). Thus, EGR2/3 are important for the expression of BCL6 in Tfh cells and the development of Tfh cells. The lack of Tfh cells was not due to a failure to activate EGR2/3-deficient CD4 T cells because EGR2/3-deficient CD4 T cells did not have defects in activation marker expression, as indicated by CD44^high^ cell frequency (Fig. 2*D*), or effector cytokine expression (Fig. 2*E*) in response to viral infection. We did not detect similar defects in Tfh cells in *CD2-Egr2*^−/−^ or *Egr3*^−/−^ mice (data not shown), suggesting an overlapping function of EGR2 and -3 in regulation of Tfh development.

##### EGR2- and EGR3-mediated Tfh Differentiation Is Important for GC Formation

**FIGURE 3.**
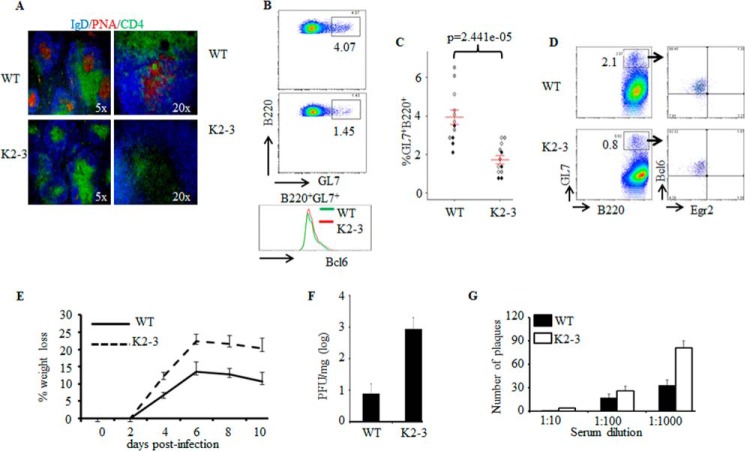
**Impaired anti-viral B cell responses and increased clinical signs in *CD2-Egr2*^−/−^*Egr3*^−/−^ mice.** WT and *CD2-Egr2*^−/−^*Egr3*^−/−^ (K2-3) mice were infected intranasally with 2 × 10^5^ pfu of vaccinia virus. *A*, splenic sections from WT and K2-3 mice 14 days postinfection were stained with PNA (*red*) and antibodies against CD4 (*green*) and IgD (*blue*). *B*, GC B cells (B220^+^GL7^+^) were analyzed in WT and K2-3 mice by flow cytometry 14 days postinfection, and the expression of BCL6 (*bottom*) was analyzed. *C*, GC B cells in these mice were quantified as a percentage of total B220 cells (gating as in *B*). *D*, EGR2 expression by B220^+^GL7^+^ cells in WT and K2-3 mice 14 days postinfection was analyzed. *E*, percentage weight loss of infected WT and K2-3 mice at the indicated time points. *F*, viral titer in lung tissue specimens from WT and K2-3 mice 8 days after infection. *G*, serum was collected from infected WT and K2-3 mice 3 weeks after infection, and the presence of antiviral antibody was assessed using a neutralization assay. The number of viral plaques in the presence of serum at the indicated dilutions is shown. Results are presented as mean ± S.E. (*error bars*) from groups of five mice of each genotype. *C*, each *symbol* represents an individual mouse from three independent experiments (indicated by *different colored symbols*); *error bars*, S.E. on either side of the mean. *, *p* < 0.0005 (Mann-Whitney two-tailed test). Data are representative of at least two independent experiments.

Tfh cells are essential for the development of GCs in response to virus infections (1, 2). In response to vaccinia virus infection, GCs were rapidly generated in spleens of wild type mice together with high levels of GL7-positive GC B cells (Fig. 3, *A–C*). However, virus infection did not induce GC formation in *CD2-Egr2*^−/−^*Egr3*^−/−^ mice, and the development of GC B cells was severely impaired (Fig. 3, *A–C*). Interestingly, BCL6 expression in GL7-positive B cells from *CD2-Egr2*^−/−^*Egr3*^−/−^ mice was largely normal compared with wild type GC B cells (Fig. 3*B*). Furthermore, we found that EGR2 was not expressed in GL7-positive B cells from virus-infected wild type mice (Fig. 3*D*), whereas *Egr3* expression was also not detected in these cells by RT-PCR (data not shown). These results suggest that defective GC formation in *CD2-Egr2*^−/−^*Egr3*^−/−^ mice results from impaired function of EGR2- and EGR3-deficient Tfh cells.

Following viral infection, *CD2-Egr2*^−/−^*Egr3*^−/−^ mice displayed more severe clinical signs than wild type counterparts (Fig. 3*E*), and viral load in the lungs of *CD2-Egr2*^−/−^*Egr3*^−/−^ mice was much higher than that in wild type counterparts (Fig. 3*F*). Consistent with the clinical pathology, the titer of neutralizing antibody was much lower in *CD2-Egr2*^−/−^*Egr3*^−/−^ mice than wild type counterparts (Fig. 3*G*), demonstrating that the generation of virus-specific antibodies was impaired in *CD2-Egr2*^−/−^*Egr3*^−/−^ mice. Thus, EGR2/3 are important for BCL6-mediated Tfh differentiation, Tfh cell-mediated GC reactions in anti-viral immune responses, and production of virus-specific antibodies that are required for effective viral clearance.

##### EGR2/3 Regulate Tfh Differentiation but Not GC B Cell Development

**FIGURE 4.**
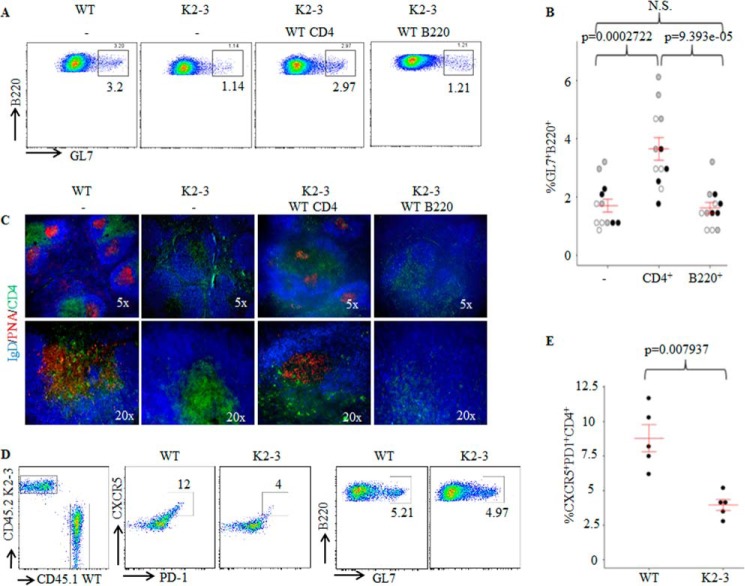
**Impaired GC formation in *CD2-Egr2*^−/−^*Egr3*^−/−^ mice results from defective Tfh cells, not GC B cell defects.** *A–C*, wild type CD4^+^ or B220^+^ cells were adoptively transferred into congenic *CD2-Egr2*^−/−^*Egr3*^−/−^ (K2-3) mice at 2 × 10^6^ cells/mouse. 24 h after transfer, mice were infected intranasally with 2 × 10^5^ pfu of vaccinia virus and analyzed 14 days postinfection. *A*, GC B cells were analyzed by flow cytometry. *B*, GC B cells in K2-3 mice (−) and K2-3 mice that received WT CD4 (CD4) or WT B220 (B220) cells were quantified as a percentage of total B220 cells. *C*, splenic sections were stained with PNA (*red*) and antibodies against CD4 (*green*) and IgD (*blue*). *D* and *E*, chimeric mice were generated by reconstitution with an equal number of bone marrow cells from wild type (CD45.1) and K2-3 mice (CD45.2). 8 weeks after reconstitution, chimeric mice were infected intranasally with 2 × 10^5^ pfu of vaccinia virus and analyzed 14 d after infection. *D*, Tfh and GC B cells were analyzed by flow cytometry after gating on CD45.1 or CD45.2 populations; Tfh cells were also gated on CD4 cells. *E*, CXCR5PD1 cells were quantified as a percentage of total CD45.1CD4 or CD45.2CD4 cells. In *B* and *E*, each *symbol* represents an individual mouse, and the data represent three independent experiments with similar results; *error bars*, S.E. on either side of the mean. *, *p* < 0.01; *N.S.*, not significant (Mann-Whitney two-tailed test). Data are representative of three independent experiments with three or four mice in each group.

EGR2 and -3 can be induced in both B and T cells by antigen receptor stimulation and function in the maintenance of B and T cell homeostasis (19). To investigate the possibility that defects in the GC reaction result from the deficiency of EGR2/3 in B cells, CD4 or B cells from wild type mice were adoptively transferred to congenic *CD2-Egr2*^−/−^*Egr3*^−/−^ mice. The recipient mice were then infected with vaccinia virus. Transfer of wild type CD4 T cells to *CD2-Egr2*^−/−^*Egr3*^−/−^ mice increased the numbers of Tfh and GC B cells and effectively restored GC formation (Fig. 4, *A–C*). In contrast, GC formation was not detected in *CD2-Egr2*^−/−^*Egr3*^−/−^ mice that received wild type B cells (Fig. 4, *A–C*). To exclude environmental differences between wild type and *CD2-Egr2*^−/−^*Egr3*^−/−^ mice, we analyzed Tfh development in mixed chimeras reconstituted with bone marrow from wild type and *CD2-Egr2*^−/−^*Egr3*^−/−^ mice. Eight weeks after bone marrow reconstitution, we did not observe enlarged peripheral lymphoid organs or overt autoimmune disease in chimeric mice. Chimeric mice had similar antiviral responses to wild type mice after virus infection. However, development of Tfh cells from EGR2- and EGR3-deficient, but not wild type, CD4 T cells was impaired (Fig. 4, *D* and *E*). In contrast, there were no differences in the development of GC B cells from wild type and EGR2/3-deficient B cells (Fig. 4*D*). These results indicate that EGR2- and EGR3-deficient T cells have an intrinsic defect in Tfh development.

**FIGURE 5.**
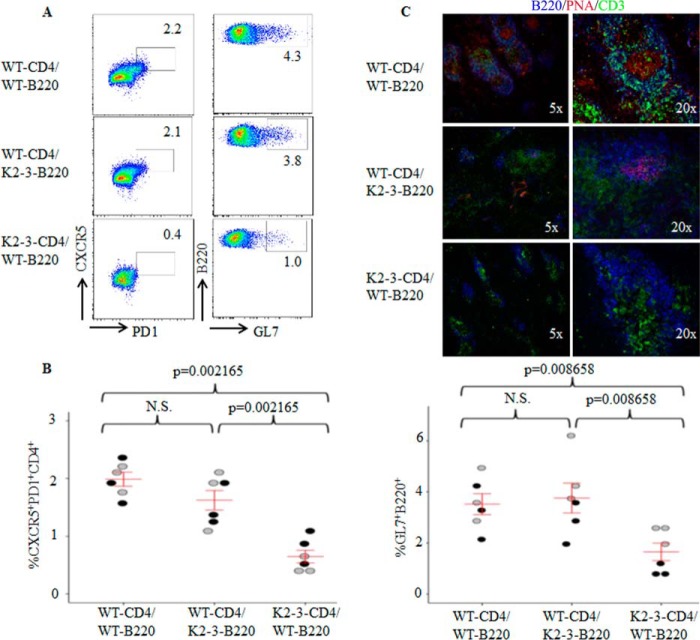
**EGR2/3-deficient CD4 T cells failed to induce GC formation in *Rag2*^−/−^ mice.** Naive CD4 and B cells were isolated from wild type and *CD2-Egr2*^−/−^*Egr3*^−/−^ mice. Wild type CD4 cells were mixed with EGR2/3-deficient B cells (WT-CD4/K2-3-B220), and wild type B cells were combined with EGR2/3-deficient CD4 cells (K2-3-CD4/WT-B220) at equal cell numbers of B and CD4 cells. Wild type CD4 cells together with wild type B cells (WT-CD4/WT-B220) served as a control. These mixtures of cells were then adoptively transferred into *Rag2*^−/−^ mice. 40 days after transfer, recipient mice were infected intranasally with 2 × 10^5^ pfu of vaccinia virus and analyzed 14 days after infection. *A*, CD4 and B220 cells from spleen and lymph nodes were gated for analysis of CD4^+^CXCR5^+^PD1^+^ Tfh cells (*left*) and B220^+^GL7^+^ GC B cells (*right*). *B*, CXCR5^+^PD1^+^ and B220^+^GL7^+^ populations were quantified as a percentage of total CD4^+^ or B220^+^ cells. *C*, splenic tissue sections were stained with anti-B220 (*blue*), PNA (*red*), and anti-CD3 (*green*). In *B*, each *symbol* represents an individual mouse from two independent experiments (indicated by *different colored symbols*); *error bars*, S.E. on either side of the mean. *, *p* < 0.01; *N.S.*, not significant (Mann-Whitney two-tailed test). Data are representative of two independent experiments with three mice in each group.

To further test whether the defective GC reaction in *CD2-Egr2*^−/−^*Egr3*^−/−^ mice is due to the function of EGR2 and -3 in B cells, we reconstituted B and T cells in *Rag2*^−/−^ mice by adoptive transfer of either wild type naive CD4 T cells in combination with EGR2/3-deficient B cells or EGR2/3-deficient naive CD4 T cells together with wild type B cells. *Rag2*^−/−^ mice that received wild type naive CD4 T cells and wild type B cells served as a control. Despite transfer of the same number of cells, the total number of B cells detected in *Rag2*^−/−^ recipient mice was only one-fifth of the number of CD4 T cells (data not shown), which is consistent with a previous report (27). However, even with few B cells, Tfh cells, GC B cells, and GC formation were detected in mice that received wild type CD4 and EGR2/3-deficient B cells (Fig. 5, *A–C*), with levels similar to those seen in mice that received wild type CD4 and wild type B cells (Fig. 5, *A–C*). In contrast, significantly fewer Tfh cells and GC B cells were observed in mice that received EGR2/3-deficient CD4 and wild type B cells (Fig. 5, *A* and *B*). Moreover, GCs were not detected in spleens from these mice (Fig. 5*C*). These results indicate that EGR2/3-deficient B cells respond normally to wild type Tfh cells for GC development. Thus, defective Tfh differentiation, but not B cell function, in *CD2-Egr2*^−/−^*Egr3*^−/−^ mice leads to the deficiency in GC formation.

##### EGR2 Directly Regulates Bcl6 Expression

**FIGURE 6.**
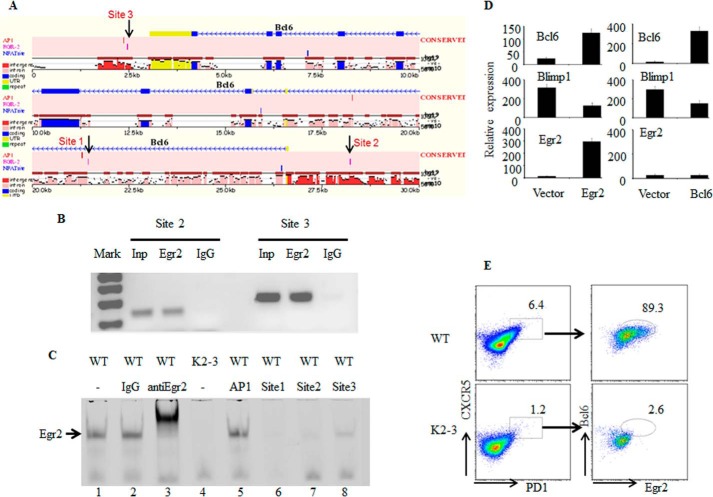
**EGR2 directly regulates *Bcl6* expression in CD4 T cells.** *A*, identification of three potential binding sites for EGR2 in conserved regions of the *Bcl6* locus using Mulan and multiTF (24). *B*, to determine whether EGR2 interacts with these sites, CD4 T cells from wild type mice were stimulated with anti-CD3 and anti-CD28 for 16 h and used in a ChIP assay with primers flanking two of the potential EGR binding sites (sites 2 and 3) in the *Bcl6* locus. Total input DNA and anti-Ig precipitates served as positive and negative controls, respectively. *C*, interaction of EGR2 with probes derived from the potential binding sites (sites 1, 2, and 3) in the *Bcl6* locus by EMSA. The EGR2 consensus binding sequence probe interacted with EGR2 from nuclear extracts of wild type CD4 T cells that were stimulated by anti-CD3 and anti-CD28 antibodies for 16 h (*lane 1*). The EGR2 probe band can be supershifted by the addition of anti-EGR2 antibody (*lane 3*) but not control IgG (*lane 2*). Probes derived from the potential EGR2 binding sites in the *Bcl6* locus (sites 1, 2, and 3) (*lanes 6–8*), but not an AP-1 probe (*lane 5*), competed for EGR2 binding. Nuclear extract from EGR2/3-deficient CD4 T cells stimulated by anti-CD3 and anti-CD28 for 16 h served as a negative control (*lane 4*). *D*, RT-PCR analysis of *Bcl6*, BLIMP1, and *Egr2* in EGR2/3-deficient CD4 T cells 24 h after transduction with control lentivirus or lentivirus encoding EGR2 (*left*) or BCL6 (*right*). *Error bars*, SD. *E*, BCL6 and EGR2 expression in Tfh cells in WT and *CD2-Egr2*^−/−^*Egr3*^−/−^ (K2-3) mice 14 days after vaccinia virus infection. Data are representative of three (*B*, *C*, and *E*) or two (*D*) independent experiments. The RT-PCR expression results are presented relative to the expression of β-actin mRNA.

The defective expression of BCL6 in EGR2/3-deficient Tfh cells suggests that EGR2/3 may regulate the expression of *Bcl6* in T cells. To investigate this possibility, we analyzed conserved regulatory regions of the *Bcl6* locus and found three potential binding sites for EGR2: one in the first intron (site 1), one in the promoter region (site 2), and one downstream of the gene (site 3) (Fig. 6*A*). Chromatin immunoprecipitation (ChIP) analysis demonstrated that EGR2 in CD4 T cells bound to two of these three sites (sites 2 and 3) (Fig. 6*B*). To further confirm the interaction of EGR2 with these sites in the *Bcl6* locus, we assessed the ability of oligonucleotides derived from these binding sites to compete with a probe containing the consensus EGR2 binding sequence for EGR2 interaction. Indeed, these oligonucleotides efficiently competed for EGR2 binding with the sequence from the promoter of *Bcl6* as the most effective competitor (Fig. 6*C*). Thus, EGR2 can directly interact with conserved elements of the *Bcl6* locus, indicating that EGR2 regulates the expression of *Bcl6* in T cells. To confirm the role of EGR2 in the expression of *Bcl6*, EGR2/3-deficient CD4 T cells were transduced with lentivirus encoding EGR2 or BCL6. Restoration of EGR2 expression effectively induced *Bcl6* expression in EGR2/3-deficient CD4 T cells (Fig. 6*D*). Interestingly, expression of BLIMP1 was reduced in EGR2/3-deficient CD4 T cells by transduction of either EGR2 or BCL6 (Fig. 6*D*), indicating that BCL6 expression is important to suppress BLIMP1 expression as described previously (28).

Following virus infection, most of the Tfh cells in wild type mice expressed BCL6, whereas BCL6 expression was defective in the few remaining Tfh cells in *CD2-Egr2*^−/−^*Egr3*^−/−^ mice (Fig. 6*E*). Moreover, the EGR2-positive Tfh cells expressed higher levels of BCL6 than did EGR2-negative cells in wild type mice (Fig. 6*E*). In contrast to BCL6, the expression levels of other genes involved in Tfh differentiation, such as *Irf4*, c-*Maf*, *Ascl2*, and *Sh2d1a* (3, 12–14, 17), were similar in wild type and EGR2/3-deficient Tfh cells (data not shown). Thus, EGR2/3 regulate Tfh differentiation by controlling *Bcl6* expression in Tfh cells.

##### Restoration of Tfh Differentiation and GC Formation in CD2-Egr2^−/−^Egr3^−/−^ Mice by EGR2 or BCL6 Transduction into EGR2/3-deficient CD4 T Cells

**FIGURE 7.**
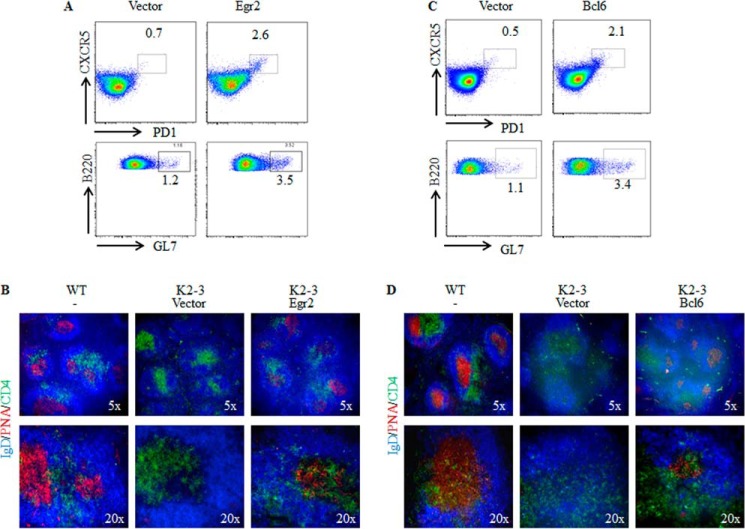
**EGR2 or BCL6 transduction rescues Tfh differentiation and GC B cell development in *CD2-Egr2*^−/−^*Egr3*^−/−^ mice.** CD4 cells isolated from *CD2-Egr2*^−/−^*Egr3*^−/−^ (K2-3) mice were transduced with EGR2-encoding (*A* and *B*) or BCL6-encoding (*C* and *D*) lentivirus. Transduced cells were isolated based on the expression of internal ribosome entry site-GFP and adoptively transferred into K2-3 mice at 1 × 10^6^ cells/mouse. 24 h after transfer, the recipient mice were infected intranasally with 2 × 10^5^ pfu of vaccinia virus and analyzed 14 days postinfection. Tfh cells and GC B cells were analyzed by flow cytometry using the indicated markers after gating on the CD4^+^ and B220^+^ populations, respectively (*A* and *C*). Splenic sections were stained with PNA (*red*) and antibodies against CD4 (*green*) and IgD (*blue*) (*B* and *D*). Data are representative of two independent experiments with three mice in each group. Adoptive transfer of K2-3 CD4 cells transduced with empty vector lentivirus served as negative controls.

To examine whether reinstated EGR2 expression or forced expression of BCL6 can restore Tfh cell function *in vivo*, EGR2/3-deficient CD4 T cells transduced with EGR2 or BCL6 were adoptively transferred into *CD2-Egr2*^−/−^*Egr3*^−/−^ mice. The recipient mice were then infected with vaccinia virus. Two weeks after infection, Tfh cells, GC B cells, and GC formation were analyzed. CD4^+^CXCR5^+^PD1^+^ cells were significantly increased in mice that received EGR2-transduced EGR2/3-deficient CD4 T cells (Fig. 7*A*). Consistent with this, transfer of EGR2-transduced EGR2/3-deficient CD4 T cells increased the percentage of GC B cells (Fig. 7*A*) and restored GC formation (Fig. 7*B*). Similarly, transfer of BCL6-transduced EGR2/3-deficient CD4 T cells increased the Tfh cell population in *CD2-Egr2*^−/−^*Egr3*^−/−^ mice and the percentage of GC B cells (Fig. 7*C*). In addition, a significant level of GCs was detected in mice that received BCL6-transduced EGR2/3-deficient CD4 T cells (Fig. 7*D*). These results demonstrate that EGR2-mediated *Bcl6* expression in Tfh cells is one of the mechanisms for Tfh cell development and function.

## Discussion

EGR2/3 have multiple functions in T cells and are essential for the control of lymphocyte homeostasis and antigen receptor-mediated proliferation (18, 19, 29, 30). However, their roles in effector T cell function are not clear. Here we have discovered a novel function of EGR2/3 in regulation of *Bcl6* expression in Tfh cells and Tfh differentiation. We found defective expression of BCL6 in EGR2/3-deficient CD4 T cells, whereas the expression of BLIMP1, a repressor of *Bcl6* (4, 28), was increased, which led to impaired Tfh differentiation and GC reactions in *CD2-Egr2*^−/−^*Egr3*^−/−^ mice. The reduced production of neutralizing antibodies and more severe clinical signs in virus-infected *CD2-Egr2*^−/−^*Egr3*^−/−^ mice, compared with wild type counterparts, demonstrated an important function of EGR2/3 in the regulation of Tfh cell-mediated B cell responses. BLIMP1 expression in EGR2/3-deficient CD4 T cells was reduced after forced expression of BCL6, suggesting that altered expression of BLIMP1 is largely due to the impaired expression of BCL6 in EGR2/3-deficient Tfh cells. Interestingly, although EGR2/3 can be induced in B cells by antigen stimulation *in vitro* (19), GC B cells did not express EGR2, and the levels of BCL6 expression in EGR2/3-deficient GC B cells were normal, suggesting that EGR2/3 are not intrinsically involved in the development of GC B cells. Our findings demonstrate a novel function of EGR2/3 in regulation of *Bcl6* expression and Tfh differentiation.

Recent studies have demonstrated that the development of Tfh cells involves at least two stages: an initial prefollicular stage and final maturation in the follicular regions (3). This paradigm is based on the findings from studies of key molecules involved in the regulation of Tfh cell chemotaxis, such as CXCR5, ICOS, and SH2D1A, and differentiation and survival, such as BCL6 (1, 3, 12, 17). CXCR5, ICOS, and SH2D1A are essential for Tfh cell migration to follicles and the initiation of Tfh differentiation (1, 3, 12, 17). CXCR5 is expressed in interfollicular Tfh cell precursors and is essential for the development of Tfh cells (3). Ascl2, a Tfh cell-specific transcription factor, has been found to induce CXCR5 expression on Tfh cell precursors, leading to migration to the follicles, where they acquire high levels of BCL6 (3). Despite the severe reduction of CXCR5^+^PD1^+^ CD4 T cell numbers in *CD2-Egr2*^−/−^*Egr3*^−/−^ mice, the expression levels of *Cxcr5*, *Icos*, and *Sh2d1a* in EGR2/3-deficient Tfh cells were normal, suggesting that EGR2/3 may not be involved in the early stage of Tfh differentiation.

BCL6 is not involved in the early stage of Tfh development, but it is the key transcription factor for both Tfh differentiation and GC B cell maturation (2, 4–6). However, the function of BCL6 in the regulation of GC B cell reactions in B cells is largely distinct from its function in Tfh cells (31). Although we do not know whether EGR2 and -3 can regulate *Bcl6* in B cells, the lack of EGR2 expression in GC B cells from wild type mice and normal levels of BCL6 in GC B cells in *CD2-Egr2*^−/−^*Egr3*^−/−^ mice indicate differences in the regulation of *Bcl6* expression in GC B and Tfh cells. BATF is one of the regulators of *Bcl6* expression in T cells and is important for Tfh differentiation by regulation of *Bcl6* and c-*Maf* expression (13). EGR2 interacts with BATF and blocks its function in suppression of AP1 activity in activated T cells (19). The enhanced BATF activity and impaired Tfh differentiation in EGR2/3-deficient CD4 T cells suggest that interaction with EGR2 does not prevent the transcriptional activity of BATF in *Bcl6* expression.

In addition to Tfh function, BCL6 controls immune homeostasis, with *Bcl6*^−/−^ mice displaying systemic inflammation associated with overproduction of Th2 cytokines (8, 32). *CD2-Egr2*^−/−^*Egr3*^−/−^ mice also develop systemic inflammation with the development of self-reactive antibodies and lupus-like disease, which partly resembles BCL6-deficient mice (8, 19, 32). Although we cannot exclude the effects of the inflammatory phenotype of EGR2- and EGR3-deficient T cells completely, the effective Tfh differentiation of wild type, but not EGR2- and EGR3-deficient, CD4 T cells in chimeric mice suggests that EGR2 and -3 have an intrinsic function in Tfh development. Furthermore, in addition to defective Tfh differentiation, the impaired expression of BCL6 in T cells may also contribute to the development of inflammatory disorders in *CD2-Egr2*^−/−^*Egr3*^−/−^ mice. Thus, similar to BCL6, EGR2 and -3 have dual function in the control of inflammation and regulation of Tfh development.

Recently, it has been found that IL-6-mediated STAT1 activation is important for *Bcl6* induction and early Tfh differentiation *in vivo* (33, 34). In addition to STAT1, STAT3 has also been found to enhance Tfh differentiation (35). However, STAT3 function in enhancing Tfh differentiation is independent of BCL6; instead, STAT3 limits Th1 differentiation through down-regulation of IL-2Ra (33). In contrast to STAT1 and STAT3, STAT5 activation induced by IL-2R signaling negatively regulates Tfh differentiation (36). However, because STAT1 and STAT3 also function in Th1 and Th17 differentiation, their roles in Tfh differentiation may depend upon factors such as when and where the stimulating cytokines are produced, whether their receptors are expressed, and also the interplay between these and other signaling pathways, including other STATS. We found that EGR2/3 indirectly control STAT1 and STAT3 activation in activated T cells by regulating SOCS1 and SOCS3 expression to prevent excessive production of Th1 and Th17 cytokines (19), suggesting that STAT1 and STAT3 have different functions in the initial activation of antigen-stimulated T cells and Tfh differentiation.

In summary, we have discovered a novel function of EGR2/3 in regulation of Tfh differentiation by controlling the expression of *Bcl6*. This EGR2/3-mediated Tfh differentiation is important for GC reactions and production of antiviral antibodies. Thus, in addition to promoting antigen receptor-induced proliferation and inhibiting the inflammatory responses of T cells, EGR2/3 are crucial for generation of high affinity antibody responses.

## Footnotes

4 The abbreviations used are:

Tfh: T follicular helper
GC: germinal center
GO: gene ontology
TRITC: tetramethylrhodamine isothiocyanate
PNA: peanut agglutinin.
